# Mutual-Attention Net: A Deep Attentional Neural Network for Keyphrase Generation

**DOI:** 10.1155/2023/8685488

**Published:** 2023-10-10

**Authors:** Wenying Duan, Hong Rao, Longzhen Duan, Ning Wang

**Affiliations:** ^1^School of Mathematics and Computer Sciences, Nanchang University, Nanchang, Jiangxi 330031, China; ^2^School of Software, Nanchang University, Nanchang, Jiangxi 330031, China

## Abstract

Neural keyphrase generation (NKG) is a recently proposed approach to automatically extract keyphrase from a document. Unlike the traditional keyphrase extraction, the NKG can generate keyphrases that do not appear in the document. However, as a supervised method, NKG is hindered by noise. In order to solve the problem that the existing NKG model does not consider denoising the source document, in this work, this paper introduces a new denoising architecture mutual-attention network (MA-net). Considering the structure of documents in popular datasets, the multihead attention is applied to dig out the relevance between title and abstract, which aids denoising. To further accurate generation of high-quality keyphrases, we use multihead attention to compute the content vector instead of Bahdanau attention. Finally, we employ a hybrid network that augments the proposed architecture to solve OOV (out-of-vocabulary) problem. It can not only generate words from the decoder but also copy words from the source document. Evaluation using five benchmark datasets shows that our model significantly outperforms the state-of-the-art ones currently in the research field.

## 1. Introduction

A keyphrase is an ordered list of words that captures the main points discussed in a natural language document [1]. Keyphrase is a significant way for people to quickly understand the key point of the document, which has been widely used in many text mining tasks, such as information retrieval, natural language processing, document summarization, and text classification [2]. Owing to public accessibility, researchers usually adopt scientific and technical publications related datasets as test platforms for keyphrase extraction algorithms. Similarly, we also use the datasets related to scientific publications to conduct keyphrase extraction [3].

Generally, existing keyphrase extraction approaches usually contain two components: keyphrase candidate search and keyphrase selection. Keyphrase candidate search is to extract a keyphrase candidate set from a document. Researchers have tried to use *N*-grams or noun phrase and compute the tightness of the inner connection in some ways to determine whether it is a phrase with independent semantics [4]. After a keyphrase candidate set is extracted, all these approaches conduct keyphrase selection to select proper keyphrases by ranking the importance of the candidate keyphrase set using different methods, either through supervised methods [5, 6] or unsupervised methods. The unsupervised method adopts the statistical feature of candidate keyphrases such as TF-IDF [7] to rank keyphrases and the unsupervised algorithm based on graph such as TextRank [8] and HITS [9, 10]. In supervised algorithms, a classifier is trained on annotated with keyphrases documents in order to determine whether a candidate phrase is a keyphrase or not.

However, the abovementioned keyphrases extraction approaches mainly have two main drawbacks. First, they are unable to extract keyphrases that do not match any contiguous subsequence of the source document (called absent keyphrases, ones that fully match a part of the text are present keyphrases). Second, they cannot capture the semantic meaning of this document. Recently, a RNN-based sequence-to-sequence framework [11] has achieved great success in sequence generation and provides an end-to-end solution to extract absent keyphrases from the source document. To overcome the abovementioned drawbacks, Meng et al. (2017) first introduced the CopyRNN [12], a RNN-based sequence-to-sequence framework, into this task [12], which incorporated a copying mechanism into the structure proposed by Gu et al. [13]. The copy mechanism is capable of solving the OOV (out-of-vocabulary) problem and allows the model to locate the important parts of the document. Different from traditional keyphrase extraction, CopyRNN can generate absent keyphrases. Therefore, we call this approach neural keyphrase generation (NKG).

A scientific publication consists of the title, abstract, and main body in general. The experimental results of supervised methods [12] indicate that using abstract instead of full text achieved better performance due to the noise in full text. The personal style of authors' writing, different vocabulary ranges, and different fields hinder the denoising. Therefore, it is a major challenge for keywords extraction that how NKG (NKG is a supervised method) can obtain high-quality keywords from the high-noise source document. Reference [12] uses the title and abstract as the source document, discarding the main body; it can denoise to some extent. However, input sequence of a neural network refers to the concatenation of the title and abstract in [12]. Usually the title represents the topics of the document; it is the least noisy and shortest sequence in the document. Although the length and noise of the abstract are shorter and lesser than the main body, it is still much longer and larger than the title. Then, the approach of Meng [12] is equivalent to concatenating a low-noise short sequence with a high-noise long sequence to obtain a high-noise long sequence.

We hypothesize that the semantics of the keyphrases and the semantics of the title are highly correlated; the relations between the abstract and the keyphrases are also important. In fact, according to our statistics on five benchmark datasets, nearly 60% of the words in the title (stop words such as a, the, and with have been removed from the title) also appear in keyphrases, thus confirming our hypothesis and statics. To overcome the above drawback, motivated by our hypothesis and statistics, we propose a novel architecture to encode the representation of the title and abstract. It takes into account the correlation between the title and keyphrases, computing the relevance between the title and abstract to denoise. Different from traditional statistical machine translation, the purpose of neural network machine translation is to establish a single neural network to maximize the translation performance through joint adjustment. The recently proposed neural machine translation model usually belongs to the category of encoder and decoder, which encodes the source statement into a fixed length vector, from which the decoder generates the translation. In addition, to further complement the informativeness of the current hidden state for next word prediction, we introduce multihead attention instead of Bahdanau attention [14]. Our model consists of three parts:

1. This is the first work to model title and abstract in a document separately and considers the relationship between them
2. Adopting multihead attention to build title-aware abstract representation and abstract-aware title representation, and self-attention to build representation of document
3. A hybrid between an attention-based RNN decoder and a pointer network to generate tokens

The key contribution of this paper is three-fold. First, this is the first work to model the title and abstract separately and consider the relationship between titles and abstracts. Second, we employ multihead attention [15] to calculate content vector instead of Bahdanau attention [14] and compute the copy distribution based on the content vector. Then, we apply a pointer-network which enables the model to copy words from the source document via pointing [16] that improves accuracy and handling of OOV words. Lastly, we apply our model to the recently-introduced KP20k dataset [12] and four other popular datasets, outperforming the current state-of-the-art neural keyphrase generation model.

The remainder of the paper is organized as follows.  Section 2 introduces related work.  Section 3 proposes mutual-attention net.  Section 4 reports our experimental results.  Section 5 gives the analysis, and then, we conclude the paper in Section 6.

## 2. Related Work

### 2.1. Encoder-Decoder Model

RNN-based encoder-decoder framework achieved state-of-the-art performances in translation task. RNN encoder-decoder is a part of the traditional phrase-based psmt system. On the basis of traditional statistical machine translation, a new joint model (RNN + psmt) is created by integrating RNN decoder-encoder and compatible with psmt. The new model is not only effective in the application of Uyghur Chinese and Chinese English machine translation but also can capture the laws of language. Bleu, an important evaluation index in machine translation, has been significantly improved [11]. However, models without attention mechanism only consider the last encoder state initializing decoder, in which case set the last encoder state as the context vector. For each decoding time step, an attention distribution is generated and the weighted sum of the encoder states is calculated as the context vector [14]. The weights of the sum are represented as attention scores which make different parts of the input sequence to be dynamically focused by the decoder during the generation of the output sequences. Subsequently, this framework achieved remarkable performance in tasks such as abstractive summarization [13, 16, 17], image caption [18, 19], and other sequence generation tasks. In the abstractive summarization task, the key information is often the low-frequency vocabulary in the corpus, even not in the vocabulary, so it cannot be recalled. Therefore, a point network is introduced to encoder-decoder framework separately [13, 16] and different copy mechanisms are proposed to solve OOV problem.

### 2.2. Neural Keyphrases Generation

There is a large body of work for extracting the present keyphrase [5, 9, 20–25]; there has been rare research on generating absent keyphrase. Meng [12] first introduces the RNN-based encoder-decoder framework to keyphrases extraction and applies the model proposed by Gu et al. [13], aiming to solve the defect that traditional approaches cannot generate absent keyphrase; it is called CopyRNN. CopyRNN outperforms popularly existing keyphrase extraction algorithms.

### 2.3. Neural Keyphrases Generation

Our copy mechanism originated in [16] is close to CopyRNN [26], but there are some small differences: we recycle the attention distribution to serve as the copy distribution, but CopyRNN uses two separate distributions. Our model can copy words from the source document, but the pointer components of CopyRNN activate only for OOV [2, 13].

### 2.4. Multihead Attention

Multihead attention, proposed by Vaswani [15], has been successfully applied to many tasks, including semantic role labeling [27] and relation extraction [28]. In this paper, we adopt the multihead attention to compute the title-match abstract representation and abstract-match title representation.

## 3. Model Analysis

We first describe the generic sequence-to-sequence attention-based model in Section 2.1 and then introduce our model in Section 2.2.

### 3.1. RNN Encoder-Decoder with Attention Mechanism

We start by briefly describing the underlying framework proposed by Bahdanau et al. [14]. In an RNN encoder-decoder with attention mechanism model, the RNN encoder reads an input sequence *X*=(*x*_1_, *x*_2_,…*x*_*T*_*X*__) into a set of hidden state vector **h**=(*h*_1_, *h*_2_ …, *h*_*T*_*X*__) by an RNN:

(1) $$\begin{array}{c} h_{1}=f\left(x_{t},h_{t-1}\right)\text{.} \end{array}$$

Also, on each step *t*, another RNN called decoder receives the word embedding of the previous word *y*_*t*−1_ (while training, this is the previous word of the reference keyphrase; at test time, it is the previous word emitted by the decoder) and has decoder state *s*_*t*_.

(2) $$\begin{array}{c} s_{t}=g\left(y_{t-1},c_{t},s_{t-1}\right)\text{.} \end{array}$$

The attention distribution *a*_*t*_ is calculated as in [14]:

(3) $$\begin{array}{c} a^{t}=softmax\left(e^{t}\right)\text{,}\\ e_{t}^{i}\propto \nu^{T}\;\tanh\left({h_{i}W}_{h}+{s_{t-1}W}_{s}+b_{attn}\right)\text{,} \end{array}$$

where *W*_*h*_, *W*_*s*_, and*b*_attn_ are the learnable parameters. Then, the attention distribution is used to compute a weighted sum of encoder hidden states, known as content vector *c*_*t*_:

(4) $$\begin{array}{c} c_{t}=\underset{i}{\sum }a_{i}^{t}h_{i}\text{.} \end{array}$$

Next, *c*_*t*_ is concatenated with the decoder state *s*_*t*_ and fed into linear layers to produce the predictive vocabulary distribution *P*_vacob_ formulated as follows:

(5) $$\begin{array}{c} P_{vacob}=softmax\left(\left(\left[s_{t};c_{t}\right]V_{1}+b_{1}\right)V_{2}+b_{2}\right)\text{,} \end{array}$$

where *V*_1_, *V*_2_, *b*_1_, and *b*_2_ are the learnable parameters. *P*_vacob_ is a probability distribution over all words in the vocabulary and provides us with our final distribution from which to predict word *y*_*t*_ at step *t*:

(6) $$\begin{array}{c} P\left(\left.y_{t}\right|\left\{y_{<t},X\right\},c_{t}\right)=P_{vacob}\text{.} \end{array}$$

Denote all the parameters to be learned in sequence-to-sequence attentional model as *θ*.

The training object is formulated as follows:

(7) $$\begin{array}{c} J\left(\theta \right)=\arg\min_{\theta }\underset{\left(X,m^{X}\right)\in D}{\sum }-\overset{T_{y}}{\underset{t=1}{\sum }}\log\;P\left(\left.y_{t}\right|y_{t-1},X;\theta \right)\text{.} \end{array}$$

### 3.2. Proposed Approach

In this subsection, we first describe the task of keyphrase generation, followed by our model in the following details:

1. The encoder of the title and abstract
2. Our mutual attention architecture
3. Hybrid decoder

#### 3.2.1. Task Description

Given a document *X*, our task is to predict a set of keyphrases *M*=(*m*_1_, *m*_2_,…,*m*_*i*_). So (*X*, *M*) is a train pair and we split (*X*, *M*) into *i* pairs: (*X*, *m*_1_^*X*^), (*X*, *m*_2_^*X*^),…, (*X*, *m*_*i*_^*X*^). Then, the model is ready to learn the mapping from source to target.

#### 3.2.2. Title and Abstract Encoder

**Figure 1:**
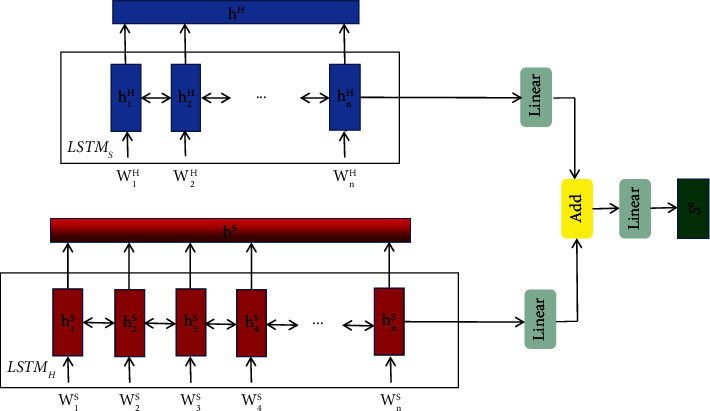
Encoder of the title and abstract.

Figure 1 shows the structure of our encoder. A document ={*w*_*t*_^*X*^}_*t*=1_^*T*_*X*_^; we first split it into title (headline) *H*={*w*_*t*_^*H*^}_*t*=1_^*T*_*H*_^ and abstract (summary) *S*={*w*_*t*_^*S*^}_*t*=1_^*T*_*S*_^. In all experimental datasets, each document *X* contains a title and an abstract. Then, the words are converted to their word-level embeddings {*e*_*t*_^*H*^}_*t*=1_^*T*_*H*_^ and{*e*_*t*_^*S*^}_*t*=1_^*T*_*S*_^. Finally, we use the bi-long short-term memory network [29] to obtain new presentation *h*^*H*^={*h*_*t*_^*H*^}_*t*=1_^*T*_*H*_^ and *h*^*S*^={*h*_*t*_^*S*^}_*t*=1_^*T*_*S*_^ of title and abstract, respectively:

(8) $$\begin{array}{c} h_{t}^{H}=Bi{LSTM}_{H}\left(e_{t}^{H},h_{t-1}^{H}\right)\text{,}\\ h_{t}^{S}=BiLSTM_{S}\left(e_{t}^{S},h_{t-1}^{S}\right)\text{.} \end{array}$$

*h*_*T*_*H*__^*H*^ and *h*_*T*_*S*__^*S*^ are the last hidden states produced by the title encoder and abstract encoder, respectively. Then, they are fed into fully connected layers to calculate the initial hidden state *s*_0_ to start the decoder.

(9) $$\begin{array}{c} s_{0}=\left(h_{T_{H}}^{H}W_{H}+h_{T_{S}}^{S}W_{S}\right)V_{3}\text{,} \end{array}$$

where *V*_3_, *W*_*H*_, and*W*_*S*_ are the learnable parameters.

#### 3.2.3. Mutual Attention

**Figure 2:**
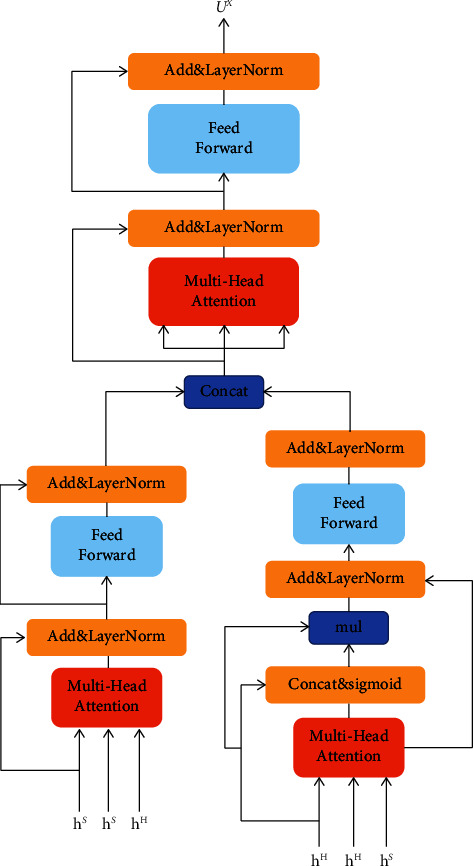
(Left) scaled dot-production attention. (Right) multihead attention.

To determine the relevance between the title and abstract, we adopt multihead attention formulation, as shown in Figure 2 by Vaswani et al. [15], to calculate title-match abstract representation *u*^*H*^={*u*_*t*_^*H*^}_*t*=1_^*T*_*H*_^, abstract-match title representation *u*^*S*^={*u*_*t*_^*S*^}_*t*=1_^*T*_*S*_^, and the representation *u*^*X*^ of document. We call it title-abstract mutual attention. The multihead attention is defined as follows:

(10) $$\begin{array}{c} MultiHead\left(Q,K,V\right)=Concat\left({head}_{1},\ldots ,{head}_{h}\right)W^{O}\text{,}\\ {head}_{i}=Attention\left(QW_{i}^{Q},KW_{i}^{K},VW_{i}^{V}\right)\text{,} \end{array}$$

where *W*_*i*_^*Q*^, *W*_*i*_^*K*^, and *W*_*i*_^*V*^ are the learnable parameters; attention refers to scaled dot product attention. Please see reference [15] for more details.

**Figure 3:**
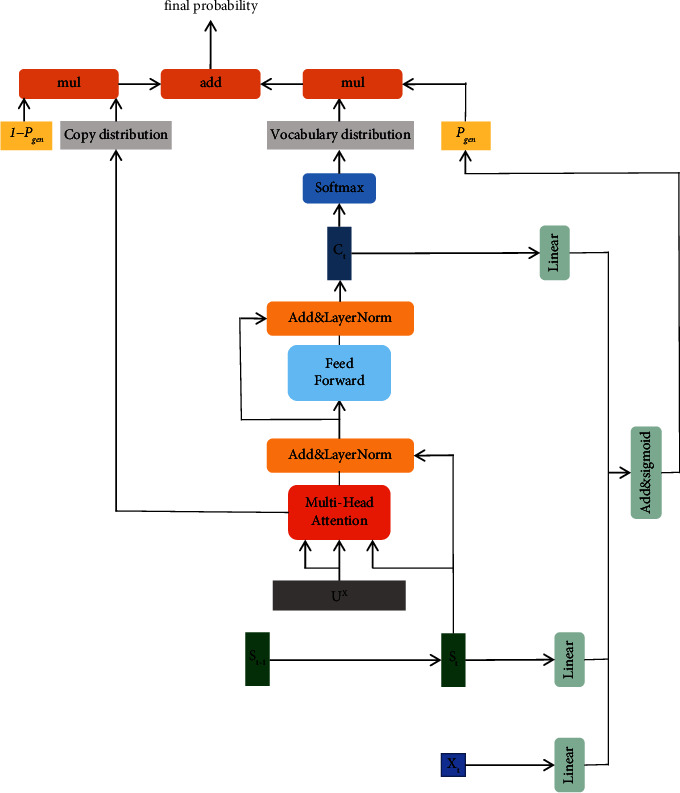
Hybrid decoder with copy mechanism multihead attention.

Figure 3 gives an overview of obtaining document representation. In addition to multihead attention layers, we use a residual block which contains a fully connected feedforward network using residual connection [30] and layer normalization [31]:

(11) $$\begin{array}{c} FFN\left(x\right)=ReLU\left(\left(xW_{1}+b_{1}\right)W_{2}+b_{2}\right)\text{,}\\ ResBlock\left(x\right)=LayerNorm\left(FNN\left(x\right)+x\right)\text{,} \end{array}$$

where *W*_2_, *W*_1_, *b*_1_, and *b*_2_ are the learnable parameters.

*(1) Computing Title-Match Abstract Representation*. We take *h*^*H*^ as queries, and *h*^*S*^ as keys and values to compute title-match abstract representation *u*^*H*^:

(12) $$\begin{array}{c} u^{H}=ResBlock\left(MultiHead\left(h^{S},h^{S},h^{H}\right)\right)\text{,} \end{array}$$

where *u*^*H*^ means that each word of the title has a corresponding weight distribution of abstract and a weighted representation of the abstract.

*(2) Computing Abstract-Match Title Representation*. It is a bit different from computing *u*^*H*^, while we compute *u*^*S*^. At each time step in the recurrent neural network, the old information will change with the current input. For longer sentences, we can imagine that the information stored in the t-k time step (*k* << T) will undergo a gradual transformation process after the *t* time step. In the process of backpropagation, the information must flow through a long time step to update the network parameters in order to minimize the loss of the network. Based on the residual block, we use a gate unit to control the flow of main body information to denoise. The abstract-match title representation *u*^*S*^ is computed as follows:

(13) $$\begin{array}{c} u^{S}=ResBlock\left(LayerNorm\left({\hat{h}}^{S}{+h}^{S}\right)\right)\text{,} \end{array}$$

where ${\hat{h}}^{S}=MultiHead\left(h^{H},h^{H},h^{S}\right)={\left\{{\hat{h}}_{t}^{S}\right\}}_{t=1}^{T_{S}},h_{t}^{S}=G_{t}{⊗h}_{t}^{S},h^{S}=concat\left(h_{1}^{S},h_{2}^{S},\ldots ,h_{T_{S}}^{S}\right)$.

(14) $$\begin{array}{c} G_{t}=\sigma \left(\left[{\hat{h}}_{t}^{S};h_{t}^{S}\right]W_{G}\right)\text{,} \end{array}$$

where *W*_*G*_ is the learnable parameter, ⊗ is the element-wise product, and *σ* is the sigmoid function.

Then, *u*^*H*^ and *u*^*S*^ are concatenated. $\bar{u^{X}}=\left[u^{H};u^{S}\right]$.

(15) $$\begin{array}{c} u^{X}=ResBlock\left(Multihead\left({\bar{u}}^{X},{\bar{u}}^{X},{\bar{u}}^{X}\right)\right)\text{.} \end{array}$$

#### 3.2.4. Hybrid Decoder

Our decoder is a hybrid between an attention-based RNN decoder and a pointer network [16, 26]. Different from [16], we use the output of encoder *u*^*X*^ and current hidden state *s*_*t*_ instead of *s*_*t*−1_ to analyze which word to copy (calculating the attention distribution *a*^*t*^ and content vector *c*_*t*_ at step *t*).

(16) $$\begin{array}{c} c_{t}=ResBlock\left(LayerNorm\left(s_{t}+MultiHead\left(u^{X},u^{X},s_{t}\right)\right)\right)\text{.} \end{array}$$

Given a fixed vocabulary *𝒱*={*v*_1_, *v*_2_,…, *v*_*N*_}, an RNN decoder can only generate words from *𝒱*. OOV words are marked as “UNK” that RNN decoder is unable to recall any keyphrases that contain “UNK.” So, we introduce a copy mechanism based on pointer components called pointer-generator network [16] to sequence-to-sequence attention-based model; it enables RNN to predict OOV by copying words from source document. In addition, for timestep *t*, we calculate the generation probability *p*_gen_ ∈ [0,1] from the context vector *c*_*t*_, decoder state *s*_*t*_, and decoder input *x*_*t*_:

(17) $$\begin{array}{c} p_{gen}=\sigma \left(c_{t}W_{c}+s_{t}W_{s}+x_{t}W_{x}+b_{ptr}\right)\text{,} \end{array}$$

where w_*c*_, *w*_*s*_, *w*_*x*_, and *b*_ptr_ are the learnable parameters and *σ* is the sigmoid function. Then, *p*_gen_ is used as a switch to choose between generating a word *y* ∈ *𝒱* from vocabulary by sampling from *P*_vacob_ or copying a word *y* ∈ *X* from the source document by sampling from the attention distribution *a*^*t*^ denoted as *P*_copy_. For word *y* in *X*, its copy probability

(18) $$\begin{array}{c} P_{copy}\left(y\right)=\underset{i:x_{i}=y}{\sum }a_{i}^{t}\text{.} \end{array}$$

So, the final probability (*y*)

(19) $$\begin{array}{c} P\left(y\right)=\left\{\begin{array}{ll} p_{gen}P_{vocab}\left(y\right)+{p_{copy}P}_{copy}\left(y\right), & y\in vocab\cap X,\\ P_{copy}\left(y\right), & y\in UNK\cap X,\\ P_{vocab}\left(y\right), & y\in vocab\;and\;y\notin X, \end{array}\right. \end{array}$$

where *p*_copy_=1 − *p*_gen_.

## 4. Experiments and Results

This section begins by experiment setup. Then, we report our result.

### 4.1. Experiment Setup

In this subsection, we first descript our benchmark datasets, followed by the baselines and evaluation metrics. Finally, we introduce the implementation details of our model.

#### 4.1.1. Datasets

We experiment with five widely used datasets in keyphrase extraction, Inspec [20], Krapivin [23], NUS [21], SemEval-2010 [25], and KP20k dataset [12]. Summary statistics of five datasets are in Table 1.

#### 4.1.2. Baseline Models

An apparatus and method for layered decoding of a dense memory array using multiple stages of a multihead decoder relates to semiconductor integrated electronics. It contains a memory array and is exactly an array that incorporates array lines with very small spacing. Specifically, it is an array with a three-dimensional memory array. The decoder structure can be advantageously used to decode word lines and/or bit lines in many different types and configurations of memory arrays. An intersection array and a NAND string memory array contain passive component memory cells (e.g., antifuse memory cells). It is especially used for memory arrays with more than one memory plane. The invention is applicable to an integrated circuit with a memory array and a method for operating the integrated circuit and the memory array and suitable for computer-readable media coding of the integrated circuit or memory array.

Our benchmarks include four unsupervised algorithms TF-IDF [24], TexRank [8], SingleRank [22], and ExpandRank [22], along with two traditional supervised algorithms KEA [6] and Maui [32] as well as the sequence-to-sequence attention-based model and CopyRNN [12].

1. TF-IDF: this is an unsupervised algorithm that uses TF-IDF scores to rank candidates and outputs the top *N*-grams as keyphrases.
2. TextRank: TextRank is a graph-based unsupervised keyword extraction algorithm that utilizes the PageRank [33] algorithm to calculate the importance of words and then ranks them according to the PageRank scores of candidate keyphrases.
3. SingleRank: it is essentially a TextRank approach with some differences.
4. ExpandRank: it is a TextRank extension that exploits neighborhood knowledge for keyphrase extraction.
5. KEA: KEA is a supervised approach. It takes TF-IDF, first occurrence, length, and node degree as features and then uses the naive Bayesian algorithm to train the model to identify whether the candidate phrase is a keyphrase. It can be either used for free indexing or for indexing with a controlled vocabulary.
6. Maui: it is an improvement of KEA that augments new features, extending the vocabulary by Wikipedia.

#### 4.1.3. Evaluation Metric

To evaluate the performance of approaches for keywords extraction, we employ F-measure (*F*_1_) to measure the models' performance on predicting the present keyphrases and recall to measure the models' performance on predicting absent keyphrases.

(20) $$\begin{array}{c} F_{1}=\frac{2\text{PR}}{P+R}\text{,} \end{array}$$

where *P* and *R* refer to precision and recall.

#### 4.1.4. Implementation Details

There are a total of 2780316 training examples. In our model, training examples are (tile, abstract, and keyphrases) triple. The training examples of other baselines are (document and keyphrases), where document refers to the concatenation of the title and abstract. The text preprocessing steps including tokenization (using Stanford CoreNLP tools [34]), lowercasing and replacing all digits with symbol “<digit>” are applied. We constructed the vocabulary with the most common 50K words, and out-of-vocabulary words were replaced with a special token “<unk>.” Each word was initialized with pretrained GloVe [35] embeddings into a vector space of 200 dimensions; the hidden size is 258 for both encoder LSTM and decoder LSTM.

We use loss on validation set to implement early stopping. Training is performed through stochastic gradient descent with the Adam optimizer [36]. The initial learning rate = 10^−1^, and gradient clipping = 2.

We train on a single Tesla K40 GPU with a batch size of 32. At test time, keyphrases are produced using a batch size of 1, beam search with beam size 200, and max depth of 6.

### 4.2. Results

We report our results of experiment in this subsection. We conduct our model on two tasks:

1. Predicting the present keyphrases
2. Predicting the absent keyphrases

#### 4.2.1. Predicting the Present Keyphrases

We evaluate the performance of our model on predicting the present keyphrases, because of which the traditional extraction models can only extract keyphrases from the source document. The result is shown in Table 2 including comparisons with our baselines. The best scores are highlighted in bold. We can see that unsupervised models (TF-IDF, TexRank, SingleRank, and ExpandRank) are more robust than traditional supervised models (Maui and KEA). But, deep neural networks are as robust as unsupervised models on all datasets. The results demonstrate that our model improves the performance over RNN and CopyRNN on all the benchmark datasets.

**Figure 4:**
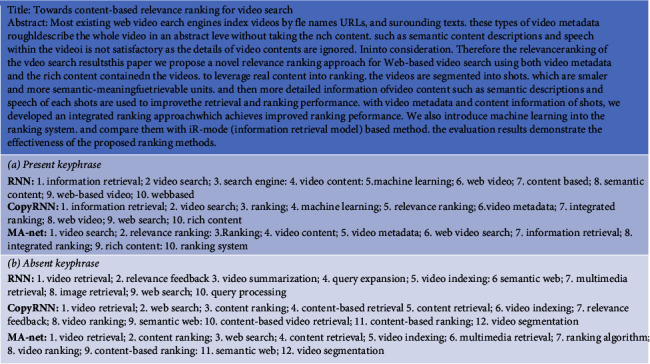
An example of result by RNN, CopyRNN, and MA-net. The ground truth is highlighted in bold.

Figure 4(a) is an example of the results of RNN, CopyRNN, and MA-net on predicting the present keyphrase. However, since neither RNN nor CopyRNN model the relationship between the title and the abstract, the phrase “information retrieval” which is not the ground truth has the highest rank and the both RNN and CopyRNN generate the phrase “machine learning” that MA-net does not generate. Although “information retrieval” and “machine learning” are related to the topic of this document, but it is too general to be selected as a keyphrase, our model predicts finer-grained keyphrases and gives them a higher ranking. Through knowledge extraction, we have obtained a large number of entities and relationships, but due to different sources, there will be a lot of noise data and duplicate data. Our model MA-net uses multihead attention to model long-term dependencies between titles and abstracts. It highlights the tile representation and the abstract representation associated with the title, reducing the noise. Therefore, the phrase “relevance ranking” contained in the title got higher ranking in MA-net than RNN and CopyRNN. The phrases that are less relevant to title such as “information retrieval” got lower ranking in MA-net than RNN and CopyRNN.

#### 4.2.2. Predicting Absent Keyphrases

As stated before, one advantage of neural keyphrase generation (NKG) is that it can predict absent keyphrases based on “understanding” of semantic information.

Only RNN and CopyRNN can handle this task. Therefore, following the previous study [12], we compare the performance of RNN, CopyRNN, and our models in terms of recall of top 10 and top 50 results. For training, we utilize both present and absent keyphrases in training datasets. For evaluating, we use the absent keyphrases in testing datasets. The results are presented in Table 3. As observed from Table 3, our model outperforms our baselines on all datasets. From Figure 4(b), we observed similar result as predicting the present keyphrase; the ranking of phrase “content based ranking” enters top 10, whereas in CopyRNN, it ranks only 34. “Video segmentation” enters top 50, whereas in CopyRNN, it ranks 64. This also benefits from the modeling of long-term dependencies between titles and abstracts.

## 5. Analysis

### 5.1. Impact of 3 Components

To further study the impact of the 3 components, we proposed the sequence-to-sequence attention-based model and we conduct a set of experiments in this subsection to compare the performance of the following model in the tasks mentioned in Section 4:

1. RNN: the sequence-to-sequence attention-based model proposed in [11]
2. CopyRNN: the model proposed in [12], which augments RNN by a copy mechanism proposed in [13]
3. RNN + pointer-generator (PG, pp. the model proposed in [16])
4. RNN + Mutual attention (MA, pp. a model based on RNN, which incorporates the proposed mutual attention)
5. RNN + multihead attention (MHA, pp. a model based on RNN, using multihead attention instead of Bahdanau attention)
6. RNN + pointer-generator + mutual attention (PG + MA, pp. a model based on RNN + pointer-generator, which incorporates the proposed mutual attention)
7. RNN + pointer-generator + multihead attention (PG + MHA, pp. a model based on RNN + pointer-generator, using multihead attention instead of Bahdanau attention)

The result of predicting the present keyphrases and absent keyphrases is shown in Tables 4 and 5, respectively. Due to space limitation, here, we report only the average F_1_@5/*F*_1_@10 on predicting the present keyphrase and *R*@10/*R*@50 on predicting the absent keyphrase.

#### 5.1.1. Comparison of RNN, CopyRNN, and PG

Here, we compare the two sequence-to-sequence attention-based models, they both have copy mechanism. As we can see, the performance of PG greatly outperformances RNN on both tasks and is comparable to CopyRNN. Explanation of Rui Meng et al. [12] for this result is that the RNN model is only concerned with finding the hidden semantics behind the document, which may tend to generate keyphrases or words that are too general and may not necessarily refer to the source. Especially, both CopyRNN and PG have copy mechanism, but the performance of PG is 0.39%/0.41% better than CopyRNN on the present keyphrases and better than CopyRNN on the absent keyphrases. We give one possible explanation for this result. As in the case of Rui Meng et al.'s study [12] and as mentioned above, RNN tends to generate generic keyphrases or words from a fixed vocabulary. The copy mechanism of CopyRNN can only copy OOV words from the document. Therefore, it is hard to handle rare but in-vocabulary words. But, our model is able to copy rare but in-vocabulary words.

#### 5.1.2. Comparison of PG, MA, and MHA

From the comparison of our proposed components (RNN + pointer-generator, RNN + mutual attention, and RNN + multihead attention), we can see RNN + pointer-generator achieves the best result on both tasks, RNN + mutual attention is slightly before RNN, but RNN + multihead attention failed to bring any benefit to RNN. This indicates that the copy mechanism is in fact a critical technic to improve the performance of automatic keyword extraction.

#### 5.1.3. Comparison of PG, PG + MA, and PG + MHA

The result is evidence that the proposed architecture can improve the performance of both tasks based on PG. This shows that our proposed method is effective.

### 5.2. Effect of Hand-Crafted Feature

We report the effect of hand-crafted features (POS tags and named-entities as well as TF and IDF) in this subsection. For discrete features such as POS tags, we use one-hot representation, for continuous features such as TF and IDF; we convert them into categorical values by discretizing them into a fixed number of bins and use one-hot representations to indicate the bin number they fall into. We try two ways to use features:

1. Method I: we concatenate features and word embedding as the input of our model
2. Method II: emerging with the representation of text: concatenating features and last dim (feature dim) of *u*^*X*^

The result of predicting the present keyphrase is shown in Table 6. Method II outperforms method I. It is clear from Table 6 that method I achieves higher *F*1 scores than method II. We offer one possible explanation for the observation. In method I, features and word embedding are the first layer of our model; the rest of the network needs to be trained from scratch. In method II, the merging of features and text representation (*u*^*X*^) directly participates in the calculation of attention, and it is easier to learn which word is important, thereby improving the accuracy of the generation.

We do not report the result of predicting absent keyphrases since both methods have no effect. We believe that, compared with predicting the present keyphrases, predicting the absent keyphrases is at a higher semantic level, requiring a better understanding of content. However, hand-crafted features are not semantic feature.

### 5.3. How Generative Is Our Model?

Our model can not only generate words from fixed vocabulary but also copy words from source document. Therefore, our model can be viewed as a balance between extraction and generation.

However, the advantage of our model on predicting the absent keyphrase is not so obvious of that on predicting the present keyphrase. We observed from Tables 2 and 3 that our model is (241.2%, 133.3%,105.3%, 87.9%, and 96.7%) higher on *F*_1_@5 and *F*_1_@10 (456.3%, 207.9%, 166.2%, 87.9%, and 47.1%) higher on *F*_1_@10 on predicting the present keyphrase than RNN (64.5%, 24.2%, 12.0%, 12.2%, and 78.3%) on recall@10 and (77.1%, 32.1%, 33.7%, 40.0%, and 57.6%) on recall@50 on predicting the present keyphrase. So, we concern that how generative is our model?

The value of the generation probability *p*_gen_ gives a measure of the generativeness of our model. During test, the model is heavily inclined to copy, the mean value of *p*_gen_ is only 0.13. This phenomenon may be the reason that the performance of predicting the present keyphrases is much better than the performance of predicting the absent keyphrases.

## 6. Conclusion

In this paper, we present a deep attentional neural network called MA-net for keyphrase generation. We introduce the multihead attention to obtain representation for the title and document and then use the pointer networks to locate the words to copy. Our model achieves state-of-the-art results on KP20k dataset and four other popular datasets. For future work, we will try to design new network structures to improve the performance of predicting absent keyphrases and consider the correlation among keyphrases.

## Figures and Tables

**Table 1:** Statistics of datasets.

Dataset	#total	#train	#validation	#test
Inspec	2000	1500	0	500
Krapivin	2034	1734	0	400
NUS	0	0	0	211
SemEval-2010	288	188	0	100
KP20k	567830	547830	20000	20000

Total: dataset size of total. Train: size of train set. Validation: size of validation set. Test: size of test set.

**Table 2:** Comparison results of predicting present keyphrases on *F*_1_ score at top 5 and top 10.

Datasets	Inspec		Krapivin		NUS		SemEval		KP20k	
Metric	*F*_1_@5	*F*_1_@10	*F*_1_@5	*F*_1_@10	*F*_1_@5	*F*_1_@10	*F*_1_@5	*F*_1_@10	*F*_1_@5	*F*_1_@10
TF-IDF	0.221	0.313	0.129	0.160	0.136	0.184	0.128	0.194	0.102	0.126
TextRank	0.223	0.281	0.189	0.162	0.195	0.196	0.176	0.187	0.175	0.147
SingleRank	0.214	0.306	0.189	0.162	0.140	0.173	0.135	0.176	0.096	0.119
ExpandRank	0.210	0.304	0.081	0.126	0.132	0.164	0.139	0.170	N/A	N/A
Maui	0.040	0.042	0.249	0.216	0.249	0.268	0.044	0.039	0.270	0.230
KEA	0.098	0.126	0.110	0.152	0.069	0.084	0.025	0.026	0.171	0.154
RNN	0.085	0.064	0.135	0.088	0.169	0.127	0.157	0.124	0.179	0.189
CopyRNN	0.278	0.342	0.311	0.266	0.334	0.326	0.293	0.304	0.333	0.262
MA-net	**0.284**	**0.356**	**0.315**	**0.271**	**0.347**	**0.338**	**0.295**	**0.311**	**0.342**	**0.278**

The best results are highlighted in bold.

**Table 3:** Comparison results of predicting the absent keyphrases on recall at top 10 and top 50.

	RNN		CopyRNN		Our model	
	*R*@10	*R*@50	*R*@10	*R*@50	*R*@10	*R*@50
Inspec	0.031	0.061	0.047	0.100	**0.051**	**0.108**
Krapivin	0.095	0.156	0.113	0.202	**0.118**	**0.206**
NUS	0.050	0.089	0.058	0.116	**0.064**	**0.119**
SemEval	0.041	0.060	0.043	0.067	**0.046**	**0.084**
KP20k	0.083	0.144	0.125	0.211	**0.148**	**0.227**

The best results are highlighted in bold.

**Table 4:** Impact of 3 components on predicting the present keyphrase.

Datasets	Inspec		Krapivin		NUS		SemEval		KP20k	
Metric	*F*_1_@5	*F*_1_@10	*F*_1_@5	*F*_1_@10	*F*_1_@5	*F*_1_@10	*F*_1_@5	*F*_1_@10	*F*_1_@5	*F*_1_@10
RNN	0.085	0.064	0.135	0.088	0.169	0.127	0.157	0.124	0.179	0.189
CopyRNN	0.278	0.342	0.311	0.266	0.334	0.326	0.293	0.304	0.333	0.262
PG	0.281	0.342	0.313	0.267	0.333	0.330	0.291	0.303	0.335	0.264
MA	0.085	0.065	0.138	0.089	0.170	0.126	0.159	0.125	0.182	0.194
MHA	0.084	0.064	0.135	0.086	0.170	0.126	0.157	0.123	0.180	0.191
PG + MA	0.288	0.352	0.315	0.269	0.344	0.337	0.293	0.309	0.349	0.276
PG + MHA	0.283	0.343	0.313	0.269	0.335	0.333	0.291	0.304	0.336	0.266

**Table 5:** Impact of 3 components on predicting the absent keyphrase.

Datasets	Inspec		Krapivin		NUS		SemEval		KP20k	
Metric	*R*@10	*R*@50	*R*@10	*R*@50	*R*@10	*R*@50	*R*@10	*R*@50	*R*@10	*R*@50
RNN	0.031	0.061	0.095	0.156	0.050	0.089	0.041	0.060	0.083	0.144
CopyRNN	0.047	0.100	0.113	0.202	0.058	0.116	0.043	0.067	0.125	0.211
PG	0.047	0.101	0.114	0.203	0.058	0.117	0.044	0.70	0.127	0.215
MA	0.032	0.066	0.099	0.160	0.053	0.090	0.042	0.062	0.088	0.151
MHA	0.031	0.060	0.095	0.157	0.051	0.093	0.040	0.61	0.085	0.147
PG + MA	0.051	0.107	0.117	0.206	0.063	0.119	0.046	0.083	0.142	0.221
PG + MHA	0.048	0.102	0.114	0.204	0.060	0.117	0.045	0.069	0.129	0.213

**Table 6:** The effect of two feature-rich methods of predicting the present keyphrases.

	Our model		Method I		Method II	
	*F*_1_@5	*F*_1_@10	*F*_1_@5	*F*_1_@10	*F*_1_@5	*F*_1_@10
Inspec	0.284	0.356	0.286	0.357	**0.290**	**0.359**
Krapivin	0.315	0.270	0.315	0.270	**0.318**	**0.272**
NUS	0.347	0.338	0.348	0.338	**0.349**	**0.341**
SemEval	0.295	0.311	0.295	0.312	**0.299**	**0.315**
KP20k	0.342	0.278	0.343	0.278	**0.346**	**0.281**

The best results are highlighted in bold.

## Data Availability

The data used to support the findings of this study are available from the corresponding author upon request.
